# Effect of a Nutraceutical-Oriented Dietary Intervention on Serum Carotenoids and Antioxidant Vitamin Concentrations in Patients with Neovascular Age-Related Macular Degeneration

**DOI:** 10.3390/antiox15070884

**Published:** 2026-07-16

**Authors:** Daria Szulim, Elżbieta Kucharska, Anna Machalińska, Leszek Kuprjanowicz, Piotr Czupryński, Małgorzata Szczuko

**Affiliations:** 1 Department of Bromatology and Nutritional Diagnostics, Pomeranian Medical University in Szczecin, 70-111 Szczecin, Poland; 2 Clinical Trials Support Center, Pomeranian Medical University in Szczecin, Ul. Unii Lubelskiej 1, 71-252 Szczecin, Poland; piotr.czuprynski@pum.edu.pl; 3 Department of Commodity Science, Quality Assessment, Process Engineering and Human Nutrition, West Pomeranian University of Technology in Szczecin, Kazimierza Królewicza St, 4, 71-550 Szczecin, Poland; elzbieta.kucharska@zut.edu.pl; 4 First Department of Ophthalmology, Pomeranian Medical University, Al. Powstańców Wielkopolskich 72, 70-111 Szczecin, Poland; anna.machalinska@pum.edu.pl (A.M.); leszek.kuprjanowicz@pum.edu.pl (L.K.)

**Keywords:** age-related macular degeneration, AMD, diet, nutritional intervention, lutein, zeaxanthin, lycopene, vitamin A, vitamin E, antioxidants, anti-VEGF therapy, carotenoids

## Abstract

Background: Age-related macular degeneration (AMD) is one of the most common causes of irreversible impairment of central vision in the elderly population. Although anti-VEGF therapy remains the standard treatment for neovascular AMD, nutritional factors may influence disease progression and retinal health. The aim of this prospective controlled study was to evaluate the effects of an individualized dietary intervention with nutraceutical characteristics on serum concentrations of lutein, zeaxanthin, lycopene, vitamin A, and vitamin E in patients with neovascular AMD receiving anti-VEGF therapy. Methods: This prospective controlled study included 43 patients with neovascular AMD who completed a six-month follow-up period. Participants were allocated to either a control group receiving anti-VEGF therapy alone or an intervention group receiving anti-VEGF therapy combined with an individualized dietary plan. The dietary intervention emphasized foods naturally rich in carotenoids, antioxidant vitamins, trace elements, dietary fiber, and omega-3 fatty acids. Serum concentrations of lutein + zeaxanthin, lycopene, vitamin A, and vitamin E were determined using liquid chromatography coupled with tandem mass spectrometry (LC-MS/MS) at baseline and after six months. Results: Serum lutein + zeaxanthin concentrations increased significantly within both groups (control 0.31 ± 0.61 to 0.49 ± 0.69 mg/L; intervention 0.51 ± 0.54 to 0.87 ± 0.89 mg/L). The increase was numerically larger in the intervention group, but the between-group difference was not statistically significant after adjustment for baseline concentrations (ANCOVA, *p* = 0.30). Lycopene increased significantly within the intervention group only (0.20 ± 0.15 to 0.43 ± 0.30 μmol/L); the between-group difference was not significant after baseline adjustment (*p* = 0.24). Vitamin A increased significantly within both groups and vitamin E within the intervention group only; however, no between-group difference remained significant after baseline adjustment (vitamin A *p* ≈ 1.00, vitamin E *p* = 0.96). The greatest increase in lutein + zeaxanthin concentrations was observed among participants with improved retinal status. These descriptive findings should be interpreted cautiously because subgroup analyses were not powered. Correlation analyses performed after six months demonstrated positive associations between serum lutein + zeaxanthin, lycopene, and vitamin A concentrations. Conclusions: An individualized dietary intervention rich in naturally occurring bioactive compounds was associated with within-group improvements in serum carotenoid and antioxidant vitamin status. After adjustment for baseline values, between-group differences did not reach statistical significance, consistent with the exploratory, non-powered pilot design. These preliminary findings, including the observed effect sizes, are intended to inform the design of an adequately powered future study rather than to establish a between-group benefit of dietary management. Particularly pronounced changes were observed for lutein + zeaxanthin and lycopene concentrations. These findings support the potential role of dietary management as an adjunct to anti-VEGF therapy. However, given the nature of the study and the relatively small sample size, larger multicenter studies are needed to confirm these observations.

## 1. Introduction

Age-related macular degeneration (AMD) is a retinal disease affecting the macula, the central part of the retina responsible for high-acuity vision. The macula enables the perception of fine details, color recognition, and contrast sensitivity. As the disease progresses, irreversible changes occur in the retina and choroid, leading to central vision loss accompanied by atrophy or detachment of the retinal pigment epithelium. Consequently, AMD may result in severe visual impairment and a substantial reduction in quality of life. AMD is recognized as the leading cause of blindness among older adults in developed countries [1].

AMD occurs in two main forms: atrophic (dry) and neovascular (wet). The atrophic form is characterized by progressive degeneration of the retinal pigment epithelium and photoreceptors with drusen formation, whereas the neovascular form involves the growth of abnormal blood vessels beneath the retina, resulting in fluid or blood leakage and rapid central vision loss if left untreated [2,3,4].

Growing evidence suggests that nutritional strategies may support standard AMD management as an adjunct, by modulating inflammatory and oxidative processes involved in disease pathogenesis [5].

A diet rich in antioxidant compounds may play a protective role in AMD by reducing oxidative stress and supporting retinal function. Among the nutrients most frequently investigated in this context are carotenoids, antioxidant vitamins, and trace elements, which may contribute to retinal protection through complementary biological mechanisms [6].

Building on these observations, the present secondary exploratory analysis investigated whether an individualized nutraceutical-oriented dietary intervention influences serum concentrations of lutein + zeaxanthin, lycopene, vitamin A, and vitamin E in patients with neovascular AMD receiving anti-VEGF therapy. Additionally, we explored whether changes in these circulating antioxidant markers were associated with retinal status, classified as improvement, stabilization, or deterioration after six months of treatment.

*Carotenoids and AMD*

Among dietary carotenoids, lutein, zeaxanthin, and lycopene are regarded as particularly relevant to retinal health owing to their antioxidant and protective properties. Humans rely entirely on dietary intake to obtain carotenoids, as these compounds are not synthesized endogenously. Because carotenoids are lipophilic compounds, their bioavailability is influenced by dietary fat intake and food processing methods [7,8].

Lutein and zeaxanthin selectively accumulate in the macula, where they form the macular pigment and protect retinal tissues by filtering blue light and reducing oxidative stress [9,10,11]. In addition, their antioxidant properties may contribute to the prevention of photoreceptor apoptosis and retinal degeneration [8,12].

The distribution of lutein and zeaxanthin may also be influenced by adipose tissue, where these lipophilic carotenoids can be stored, potentially reducing their retinal availability. Furthermore, visceral adipose tissue promotes chronic low-grade inflammation through cytokines such as IL-6 and TNF-α, which have been implicated in AMD pathogenesis [13,14,15,16].

Dietary intake of lutein and zeaxanthin can be increased through the consumption of green leafy vegetables, such as kale, spinach, and beet greens, as well as yellow and orange vegetables, including corn and bell peppers [17,18].

Lycopene is another carotenoid of particular interest in AMD because of its potent antioxidant and anti-inflammatory properties [19,20]. Although it does not accumulate in the macula, it may indirectly support retinal health by reducing oxidative stress and protecting macular carotenoids from oxidative damage [21,22].

Major dietary sources of lycopene include tomatoes and tomato-based products, such as tomato sauce, paste, and puree, as well as watermelon and red grapefruit [22,23].

*Antioxidant Vitamins in AMD*

Oxidative stress is considered one of the key mechanisms involved in AMD pathogenesis. Vitamins A and E play complementary roles in protecting retinal tissues against oxidative damage [6,24]. Vitamin A is essential for normal visual function, as its active form, retinal, is a key component of rhodopsin and is required for phototransduction in rod photoreceptors. Whereas vitamin E protects retinal cell membranes against lipid peroxidation and may contribute to the modulation of inflammatory processes, which are recognized as important factors in AMD progression [25,26,27].

Major dietary sources of vitamin A include liver, carrots, sweet potatoes, and spinach [27]. Rich sources of vitamin E include nuts (particularly almonds), seeds (especially sunflower seeds), and green leafy vegetables [28].

Besides vitamins A and E, vitamin C also plays an important role in retinal antioxidant protection through its ability to neutralize reactive oxygen species and support cellular defense against oxidative stress [6]. Vitamin C is abundant in citrus fruits, such as oranges and grapefruits, as well as broccoli, bell peppers, and strawberries [28].

## 2. Materials and Methods

### 2.1. Study Population

This study presents an additional analysis of data obtained from a prospective dietary intervention conducted among patients with neovascular age-related macular degeneration (AMD). The study was performed at the First Department of Ophthalmology, Pomeranian Medical University, Szczecin, Poland, and included patients aged ≥65 years receiving intravitreal bevacizumab therapy. A total of 43 participants completed the six-month follow-up and were included in the present analysis, comprising 22 participants in the intervention group and 21 participants in the control group. All patients had clinically confirmed neovascular AMD and received intravitreal anti-VEGF therapy (off-label bevacizumab, as used at the study center) throughout the study period. All participants were of White (Caucasian) European ethnicity, consistent with the local single-center population; this supports the comparison of serum lycopene and lutein + zeaxanthin concentrations with published Caucasian reference values. Participants were offered participation in either the anti-VEGF therapy alone group or the anti-VEGF therapy plus individualized dietary intervention group and were assigned according to their preference. No individualized dietary recommendations were provided to the control group beyond standard clinical care.

Ophthalmological assessment included clinical examination, optical coherence tomography (OCT), and OCT angiography (OCTA) using the Spectralis OCT/OCTA system (Heidelberg Engineering, Heidelberg, Germany). Retinal status was evaluated by the same ophthalmologist and classified as improvement, stabilization/no progression, or deterioration after six months of follow-up. Retinal status was assessed by the same ophthalmologist based on the combined interpretation of clinical examination and OCT/OCT-angiography findings, including changes in central retinal thickness, intraretinal and subretinal fluid, retinal morphology, and features of macular neovascularization, and classified after six months as improvement (1), stabilization/no progression (0), or deterioration (−1) [29].

Detailed information regarding patient recruitment, eligibility criteria, study design, ophthalmological assessment, and ethical approval has been published previously [29]. The present manuscript reports additional findings from the same study, focusing on serum concentrations of carotenoids, antioxidant vitamins, and selected biochemical markers that were not included in the previous publication.

This work is a secondary, exploratory analysis of a single-center pilot (feasibility) study rather than a clinical trial. Because of its exploratory nature and the absence of reliable prior effect-size data for the proposed whole-food dietary intervention, no formal a priori sample-size estimation was performed. The sample size was determined by feasibility, including the availability of eligible patients, the organizational and financial capacity of the center, and time constraints related to a doctoral project. The primary aims were to assess feasibility and to obtain preliminary effect-size estimates to inform an adequately powered future study, in which a significance level of α = 0.05 and a power of 1 − β = 0.80 will be adopted and the sample size calculated for a χ^2^ test comparing two proportions, with therapeutic success defined as retinal improvement on OCT.

### 2.2. Dietary Intervention

**Figure 1:**
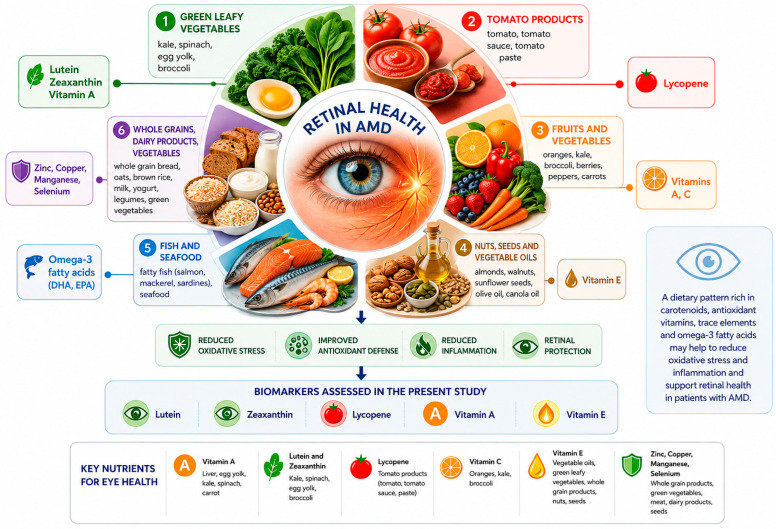
Conceptual framework of the retinal-supportive dietary intervention used in patients with neovascular AMD.

Participants followed an individualized dietary intervention for six months. Dietary plans were developed using dedicated nutritional software (TiqDiet Polska Sp. z o.o., Szczecin, Poland; https://tiqdiet.com; accessed on 1 January 2026) and tailored to age, sex, nutritional status, comorbidities, food intolerances, allergies, food preferences, and individual energy requirements. The intervention was based on a whole-food dietary approach emphasizing foods naturally rich in carotenoids, antioxidant vitamins, trace elements, dietary fiber, and omega-3 fatty acids (Figure 1).

The dietary model was designed to provide nutrients potentially relevant to retinal health, including lutein and zeaxanthin (6 mg), lycopene (5–10 mg), vitamin E (8 mg/day for women, 10 mg/day for men), zinc (8 mg/day for women, 11 mg/day for men), copper (0.9 mg/day), manganese (1.6–1.8 mg/day for women, 2.1–2.3 mg/day for men), vitamin C (75 mg/day for women, 90 mg/day for men), omega-3 fatty acids (EPA + DHA, 250–500 mg/day), selenium (55 µg/day), and vitamin A (700 µg/day for women, 900 µg/day for men) [17,30,31,32,33]. Particular emphasis was placed on the consumption of green leafy vegetables, tomato products, fruits, nuts, seeds, whole grains, and fish.

Although dietary plans differed in total energy content and macronutrient composition according to individual requirements, all variants were based on the same nutritional model and were designed to provide comparable amounts of nutrients associated with retinal protection. Participants received individualized meal plans, dietary counseling, recipes, and practical instructions regarding food preparation and portion sizes.

Adherence to the dietary intervention was monitored during regular follow-up consultations and monthly clinical visits, based on participants’ reports regarding compliance with the prescribed meal plans.

Detailed information regarding meal plan development, nutrient composition, and adherence monitoring has been reported previously [29].

### 2.3. Determination of Serum Concentrations of Vitamins A and E and Carotenoids (Lutein, Zeaxanthin, and Lycopene)

The concentrations of vitamins A and E, as well as lutein, zeaxanthin, and lycopene, were determined at the laboratory of Altium International Sp. z o.o. (formerly Perlan Technologies Polska Sp. z o.o.), Gdynia, Poland. Liquid chromatography coupled with tandem mass spectrometry (LC-MS/MS) was used. Analyses were performed using an Agilent 1290 liquid chromatograph and an Agilent 6470 mass spectrometer (Agilent Technologies Inc., Santa Clara, CA, USA). The liquid chromatograph consisted of a binary pump, an autosampler, and a column thermostat. Analyses were carried out using an InfinityLab Poroshell 120 EC-C18 column (3.0 × 100 mm, 2.7 μm; Agilent Technologies Inc., Santa Clara, CA, USA).

The mobile phase consisted of 0.1% formic acid in water and 0.1% formic acid in methanol (gradient method) at proportions of 20:80 (0.0 min) and 0:100 (5 min). The total run time was 17 min, and the column equilibration time before the next analysis was 4 min. The mobile phase flow rate was 0.5 mL/min, and the injection volume was 4 μL. Method calibration was performed using a mixture of standards: trans-β-apo-8-carotenal (Sigma-Aldrich, Darmstadt, Germany), lycopene (Merck, Darmstadt, Germany), α-tocopherol-d6 (Toronto Research Chemicals, Toronto, ON, Canada), DL-α-tocopherol (Dr. Ehrenstorfer GmbH, Augsburg, Germany), retinol (ChromaDex, Irvine, CA, USA), and lutein (Merck, Darmstadt, Germany), prepared in ethanol (Merck, Darmstadt, Germany). Trans-β-apo-8-carotenal and α-tocopherol-d6 were used as internal standards.

The concentration of lycopene was determined relative to the concentration of the internal standard β-apo-8-carotenal. The presence of the analyte in the patient sample was confirmed based on its retention time relative to the reference standard and the characteristic MRM transitions for lycopene: 536.7 → 69.1, 536.7 → 144.8, 537.5 → 144.8, and 537.5 → 69.1.

Aliquots of 200 μL of serum were transferred into 2 mL Eppendorf tubes, followed by the addition of 20 μL of a mixture of internal standards prepared in ethanol containing BHT. The mixture was vortexed for 10 s. Subsequently, 400 μL of ethanol containing 0.04% BHT was added and vortexed for 10 s, followed by the addition of 800 μL of hexane containing 0.04% BHT and vortexing for 3 min. The samples were centrifuged at 3500× *g* for 10 min at 10 °C. From each sample, 700 μL of the supernatant was transferred to a glass autosampler vial and evaporated under a nitrogen atmosphere. The residue was then reconstituted in 200 μL of ethanol containing BHT. Finally, the samples were transferred into glass inserts [34].

Under the chromatographic conditions applied, lutein and zeaxanthin exhibited identical retention times and therefore were quantified as the sum of both compounds, as reported in previous studies [35,36,37,38].

### 2.4. Statistical Analysis

Statistical analyses were performed using Statistica (version 13.1; TIBCO Software Inc., Palo Alto, CA, USA). The significance level was set at α = 0.05.

The normality of data distribution was assessed using the Shapiro–Wilk test. Variance homogeneity was assessed using Levene’s test. Depending on the distribution of the data, parametric or non-parametric tests were applied.

Student’s *t*-test was used for the analysis of normally distributed variables. The Wilcoxon signed-rank test was applied for comparisons between baseline and follow-up measurements within the same patients, whereas the Mann–Whitney U test was used for comparisons between independent groups.

Correlations between anthropometric parameters were assessed using Spearman’s rank correlation coefficients (*p*-value < 0.05 was considered statistically significant).

Box-and-whisker plots were used to visualize data distributions, interquartile ranges, and potential outliers for the analyzed biochemical parameters at baseline and after six months of follow-up.

For between-group comparisons of follow-up concentrations, analysis of covariance (ANCOVA) was performed with the baseline concentration as covariate and group as the between-subjects factor; homogeneity of regression slopes was verified, and results were confirmed with a non-parametric test (Mann–Whitney U on change scores) where residuals were non-normal.

## 3. Results

The final analysis included 43 patients with neovascular age-related macular degeneration who completed the six-month follow-up period. Baseline demographic and biochemical characteristics of the study participants are presented in Table 1. Participants were allocated to either a control group receiving anti-VEGF therapy alone or an intervention group receiving anti-VEGF therapy combined with an individualized dietary intervention. In both groups, the mean age exceeded 65 years, and women constituted the majority of participants.

*Lutein and Zeaxanthin*

Serum lutein + zeaxanthin concentrations increased significantly during the six-month follow-up in both study groups (Table 1). In the control group, the median concentration increased from 0.31 mg/L at baseline to 0.49 mg/L after six months. A more pronounced increase was observed in the intervention group, where concentrations increased from 0.51 mg/L to 0.87 mg/L. Baseline lutein + zeaxanthin concentrations were significantly higher in the intervention group compared with the control group (0.51 vs. 0.31 mg/L).

As no established reference range exists for serum lutein and zeaxanthin concentrations, the values reported by Stimpson et al. (13.79–20.55 μg/dL; 0.14–0.21 mg/L) were used as a reference for comparison [35]. In the present study, serum concentrations exceeded this range in both groups.

**Figure 2:**
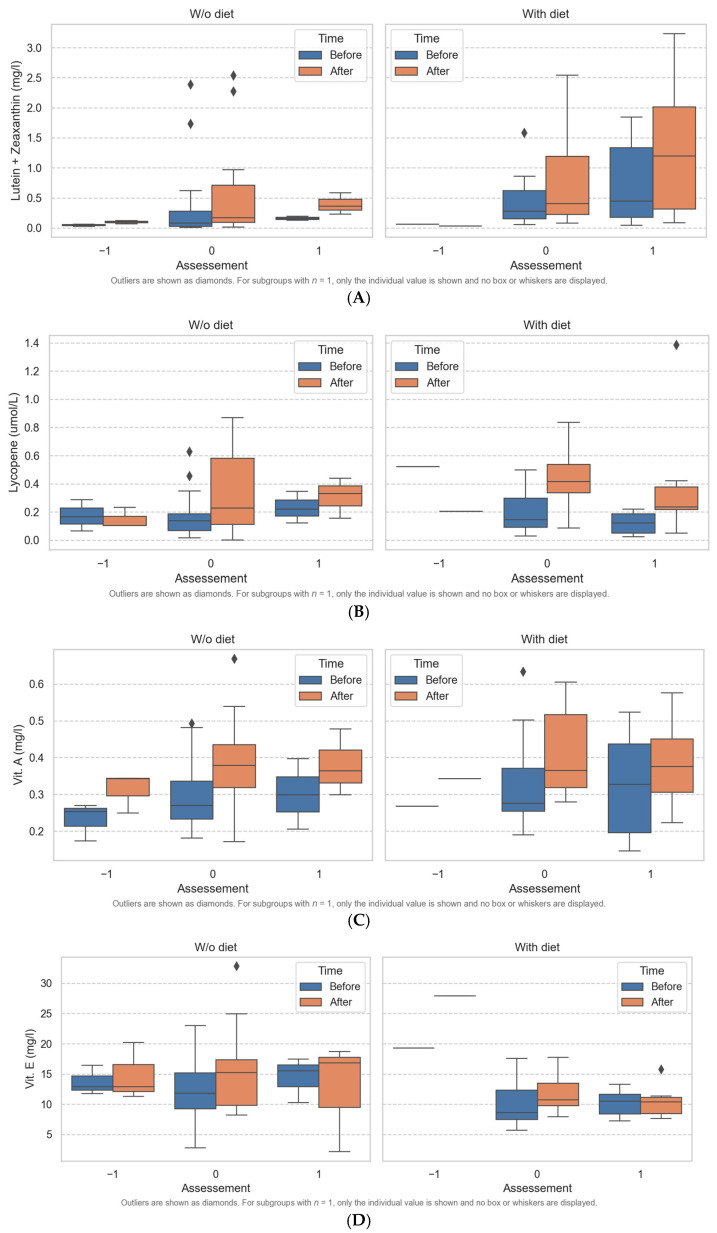
(**A**–**D**). Changes in serum concentrations of (**A**) lutein + zeaxanthin, (**B**) lycopene, (**C**) vitamin A, and (**D**) vitamin E, each shown for the control (“W/o diet”) and intervention (“With diet”) groups, stratified by retinal outcome (−1 deterioration, 0 no change, +1 improvement), before and after six months.

When stratified according to retinal outcome, the largest increase in lutein + zeaxanthin concentrations was observed among participants in the intervention group who demonstrated retinal improvement, with median values increasing from 0.45 mg/L to 1.20 mg/L (Figure 2A). More moderate increases were observed among participants with stable retinal status, whereas no increase was observed in the subgroup with retinal deterioration. In the control group, median lutein + zeaxanthin concentrations increased across all retinal outcome categories, although the magnitude of change was smaller than that observed in participants receiving the dietary intervention.

*Lycopene*

Serum lycopene concentrations were compared between the control and intervention groups. A statistically significant increase in lycopene concentration was observed in the intervention group after six months of dietary intervention, with median values increasing from 0.20 μmol/L at baseline to 0.43 μmol/L at follow-up. No statistically significant changes were observed in the control group (Table 1).

When stratified according to retinal outcome, median lycopene concentrations increased among participants with retinal improvement and stable retinal status in both study groups (Figure 2B). In contrast, a decrease in lycopene concentration was observed among participants whose retinal condition deteriorated. In the control group, median lycopene concentrations changed from 0.12 to 0.24 μmol/L in patients with retinal improvement and from 0.15 to 0.42 μmol/L in those with stable retinal status, whereas a decrease from 0.52 to 0.21 μmol/L was observed in patients with retinal deterioration. Similar trends were observed in the intervention group, with increases in participants with retinal improvement (0.22 to 0.33 μmol/L) and stable retinal status (0.14 to 0.23 μmol/L), and a decrease among those with retinal deterioration (0.17 to 0.11 μmol/L).

Reference ranges for serum lycopene concentrations have not been established. Therefore, the results were compared with values reported by Mayne et al. for Caucasian populations (0.34–0.88 μmol/L) [39]. Baseline median lycopene concentrations were generally below this range, whereas higher concentrations were observed after six months, particularly among participants receiving the dietary intervention.

*Vitamin A*

Serum vitamin A concentrations increased significantly over the six-month follow-up period in both study groups. In the control group, the median concentration increased from 0.29 mg/L at baseline to 0.37 mg/L after six months, while in the intervention group it increased from 0.33 mg/L to 0.40 mg/L (Table 1). Despite the observed increases within both groups, no statistically significant differences were found between the control and intervention groups. Boxplot analysis additionally demonstrated an upward shift in median vitamin A concentrations in both groups regardless of retinal status (improvement, stabilization, or deterioration) (Figure 2C). The reference range for serum vitamin A concentrations has been reported as 30–80 μg/dL (0.3–0.8 mg/L) [40].

*Vitamin E*

A statistically significant difference in serum vitamin E concentration was observed between the control and intervention groups at baseline. Patients in the control group exhibited higher vitamin E concentrations (12.88 mg/L) compared with those in the intervention group (10.43 mg/L). During the six-month follow-up, a significant increase in serum vitamin E concentration was observed in the intervention group, rising from 10.43 mg/L to 12.33 mg/L. No significant changes were observed in the control group (Table 1).

When vitamin E concentrations were analyzed according to retinal outcome, different patterns were observed among the subgroups (Figure 2D). In the control group, median vitamin E concentrations increased in patients with retinal improvement and in those with stable retinal status, whereas little change was observed among patients whose retinal condition deteriorated. In the intervention group, median vitamin E concentrations remained relatively stable in patients with retinal improvement, increased among those with stable retinal status, and showed the greatest increase in the subgroup with retinal deterioration. However, these findings should be interpreted with caution due to the small number of participants in some retinal outcome categories. The reference range for serum vitamin E concentration in adults is 5–18 mg/L [40].

*Subgroup Analysis*

**Figure 3:**
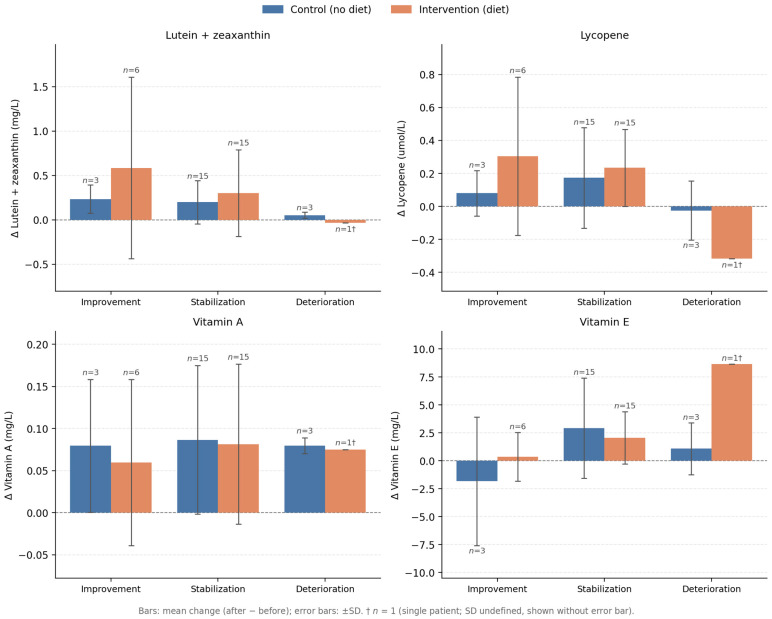
Changes in serum concentrations of lutein + zeaxanthin, lycopene, vitamin A, and vitamin E according to retinal outcome (improvement, stabilization, deterioration) after six months of anti-VEGF therapy. Values are presented as mean ± SD; error bars indicate standard deviation, and the number of participants in each subgroup is shown.

To facilitate interpretation of the subgroup analyses, the corresponding numbers of participants and measures of variability are presented in Figure 3. In the intervention group, larger increases in lutein + zeaxanthin and lycopene were observed among participants with retinal improvement than among those with stabilization or deterioration. However, because variability was high, subgroup sizes were small (particularly the deterioration subgroup, n = 1), and the error bars overlapped substantially, these findings should be regarded as descriptive and hypothesis-generating only. No consistent pattern was observed for vitamin A or vitamin E.

*Correlation Analysis*

Correlation analyses performed after six months of follow-up demonstrated positive associations between serum lutein + zeaxanthin, lycopene, and vitamin A concentrations. Participants with higher lutein + zeaxanthin concentrations also tended to exhibit higher lycopene and vitamin A levels. Similar associations were observed between lycopene and vitamin A concentrations.

*ANCOVA analysis*

After adjustment for baseline concentrations (ANCOVA), no statistically significant between-group differences were observed for any of the four markers (lutein + zeaxanthin *p* = 0.30, lycopene *p* = 0.24, vitamin A *p* ≈ 1.00, vitamin E *p* = 0.96); non-parametric analysis of change scores yielded concordant results. The larger increases observed in the intervention group for lutein + zeaxanthin and lycopene were therefore numerical and did not reach statistical significance after accounting for baseline values.

## 4. Discussion

The present study evaluated the effects of a six-month individualized dietary intervention on serum concentrations of selected carotenoids and antioxidant vitamins in patients with neovascular AMD receiving anti-VEGF therapy. The main findings indicate significant within-group increases in serum lutein + zeaxanthin, lycopene, and vitamin E in the intervention group; after adjustment for baseline values, however, none of the between-group differences reached statistical significance. In addition, the highest lutein + zeaxanthin concentrations after six months were observed among participants who demonstrated improvement in retinal status.

Although participants with retinal improvement tended to show greater increases in serum lutein + zeaxanthin and lycopene concentrations, these subgroup analyses were exploratory only. The large variability, substantial overlap of the error bars, and the very small number of participants in the deterioration subgroup preclude firm conclusions regarding the relationship between retinal outcome and changes in circulating antioxidant concentrations. These observations should therefore be considered hypothesis-generating and require confirmation in adequately powered studies.

Lutein and zeaxanthin are the principal carotenoids of the macular pigment and play an important role in retinal protection through blue-light filtration and antioxidant activity. In the present study, serum lutein + zeaxanthin concentrations increased significantly within both groups; the increase was numerically larger in the intervention group, although this between-group difference was not statistically significant after baseline adjustment (ANCOVA, *p* = 0.30). Furthermore, among patients with retinal improvement, the intervention group demonstrated the greatest increase in median lutein + zeaxanthin concentration, from 0.45 mg/L to 1.20 mg/L. These findings suggest that increased dietary intake of lutein- and zeaxanthin-rich foods may contribute to improved carotenoid status in patients with AMD. Similar observations have been reported in previous studies demonstrating increased circulating xanthophyll concentrations following dietary modification or supplementation [41,42].

An additional observation was the positive association between lutein + zeaxanthin and vitamin A concentrations. Although lutein and zeaxanthin are not precursors of vitamin A, these compounds frequently occur in the same food sources as provitamin A carotenoids. Therefore, higher concentrations of these nutrients may reflect greater consumption of vegetables and other carotenoid-rich foods, which constituted an important component of the dietary model used in the present study [43,44].

The relationship between dietary carotenoid intake, circulating carotenoid concentrations, and retinal outcomes is likely influenced by multiple factors, including lifestyle, nutritional status, and individual biological variability [45,46].

Lycopene increased significantly within the intervention group only, whereas no significant within-group change was seen in controls; the baseline-adjusted between-group comparison was, however, not significant (*p* = 0.24). After six months, serum lycopene concentrations increased from 0.20 μmol/L to 0.43 μmol/L, whereas no statistically significant change was observed in the control group. Moreover, lycopene concentrations increased among patients with retinal improvement and stable retinal status, while lower values were observed among participants with retinal deterioration. These findings may support the potential role of lycopene as a systemic antioxidant contributing to retinal protection. Previous studies have shown that lycopene effectively scavenges reactive oxygen species and may protect biological tissues against oxidative damage [19].

The correlation analyses additionally demonstrated moderate positive associations between lutein + zeaxanthin, lycopene, and vitamin A concentrations. The observed associations may reflect the common dietary sources of these compounds, particularly vegetables and fruits rich in carotenoids and antioxidant nutrients.

The observed increases in serum carotenoid concentrations may also be related to the combined intake of multiple antioxidant compounds provided by the dietary intervention. Experimental evidence suggests that mixtures of carotenoids may be more effective in reducing oxidative damage than individual compounds, indicating potential synergistic interactions between these bioactive nutrients [47].

Reference [29] reported the effects of this intervention on body composition and clinical retinal outcomes in the same cohort. The present study reports a separate set of outcomes—serum concentrations of lutein + zeaxanthin, lycopene, and vitamins A and E—that were not analyzed previously, and examines how these change with the intervention and how they relate to retinal status. In our previous analysis of this cohort, the intervention was associated with reductions in body weight, BMI, and related parameters [29]. Because carotenoids are stored in adipose tissue, such changes in body composition may plausibly contribute to the increases in circulating carotenoids observed here; however, no direct associations between carotenoid concentrations and anthropometric measures were detected in the present data, and this interpretation remains speculative given the small sample.

Vitamin A plays a fundamental role in visual function as an essential component of rhodopsin, the visual pigment responsible for vision under low-light conditions. Vitamin A deficiency has been associated with impaired dark adaptation and an increased risk of retinal dysfunction. Vitamin A is essential for the visual cycle and photoreceptor function, which may explain the clinical relevance of maintaining adequate serum concentrations in patients with AMD [48].

Serum vitamin A concentrations increased significantly in both groups during follow-up, although no significant differences were observed between the intervention and control groups. Moreover, vitamin A concentrations remained within the reference range throughout the study. These findings suggest that the dietary intervention did not substantially alter vitamin A status beyond normal physiological levels. Similar vitamin A concentrations have been reported previously in patients with AMD [49,50].

Vitamin E is highly concentrated in the retinal pigment epithelium and photoreceptor outer segments, where it plays an important role in protecting retinal tissues against oxidative damage. Experimental studies have shown that vitamin E deficiency may lead to photoreceptor degeneration [51].

A significant within-group increase in vitamin E was observed only in the intervention group. It should be noted, however, that the mean magnitude of change was similar in the two groups and the baseline-adjusted between-group difference was not significant (*p* = 0.96); this within-group result therefore does not indicate a between-group advantage of the intervention. Nevertheless, vitamin E concentrations remained within the accepted reference range both before and after the intervention. Although vitamin E is recognized as an important lipid-soluble antioxidant involved in retinal protection, the relatively short duration of follow-up may have limited the ability to detect clinically meaningful effects on retinal outcomes. Previous long-term observational studies have suggested that protective effects of vitamin E-rich diets may become apparent only after several years of sustained dietary exposure [52,53].

Although nutritional approaches to AMD have traditionally focused on dietary supplementation, particularly the AREDS and AREDS2 formulations, increasing evidence suggests that overall dietary patterns may also play an important role in retinal health [5,54,55,56,57]. The nutritional intervention adopted in this study emphasized a whole-food dietary pattern naturally enriched in carotenoids, antioxidant vitamins, trace elements, and omega-3 fatty acids. By prioritizing dietary sources rather than isolated supplementation, the intervention aimed to deliver a combination of bioactive compounds that may act synergistically to support retinal health. This concept is consistent with several principles of the Mediterranean diet, which has been linked to reduced oxidative stress and chronic inflammation [58,59,60,61].

The Mediterranean dietary pattern is characterized by a high intake of vegetables, fruits, and other plant-derived foods that provide carotenoids, including lutein, zeaxanthin, and lycopene. These compounds have been suggested to support retinal health through their antioxidant, anti-inflammatory, and light-filtering properties. Previous studies have reported associations between greater adherence to the Mediterranean diet and a lower risk of AMD progression. Given the observed increases in serum lutein + zeaxanthin and lycopene concentrations in our study, dietary patterns rich in these carotenoids may contribute to improved antioxidant status and potentially support retinal health. However, due to the relatively small sample size and the exploratory nature of the present study, these findings should be interpreted with caution and require confirmation in larger prospective studies [61].

The strengths of the present study include the prospective design, individualized dietary intervention, six-month follow-up period, and the use of LC-MS/MS methodology for biochemical assessment. The present dietary intervention differs fundamentally from the AREDS2 supplementation model. While AREDS2 provides pharmacological doses of selected nutrients through supplementation, our intervention was based on whole foods naturally rich in carotenoids, antioxidant vitamins, trace elements, and omega-3 fatty acids. Despite substantially lower nutrient doses compared with AREDS2 formulations, significant increases in serum lutein + zeaxanthin and lycopene concentrations were observed, suggesting that food-based dietary strategies may effectively improve antioxidant status in patients with neovascular AMD.

In addition to the AREDS formulation, other nutritional strategies have also been investigated in patients with neovascular AMD. Josifova et al. demonstrated beneficial effects of Ocufolin Forte supplementation administered as an adjunct to anti-VEGF therapy. In contrast, the present study evaluated a whole-food dietary intervention designed to increase the intake of carotenoids and antioxidant nutrients through habitual food consumption rather than supplementation. Despite these different nutritional approaches, both studies support the concept that nutritional interventions may complement anti-VEGF therapy by improving antioxidant status in patients with AMD [62].

Several limitations should also be acknowledged. First, the sample size was relatively small, particularly after stratification according to retinal outcomes. Second, the study was conducted at a single center. The relatively small sample size, particularly after stratification according to retinal outcomes, should be considered when interpreting the results. The subgroup analyses stratified by retinal outcome are descriptive and hypothesis-generating only. Although participants with retinal improvement tended to show greater increases in serum lutein + zeaxanthin and lycopene concentrations, the number of participants per outcome category was small and unequal (improvement: *n* = 6 intervention, *n* = 3 control; stabilization: *n* = 15 and *n* = 15; deterioration: *n* = 1 and *n* = 3). Consequently, the results for the smallest subgroups, particularly the single intervention-group patient with retinal deterioration, should be interpreted with caution. These analyses were not statistically powered, no formal between-subgroup testing was performed, and the corresponding patterns should be interpreted with caution and confirmed in larger studies.

Because of the limited sample size, multivariable models adjusting for potential confounders such as sex, baseline BMI, and comorbidities were not fitted, as they would have risked overfitting; the associations reported here are therefore unadjusted and should be interpreted as exploratory. Confounder-adjusted analyses are planned for an adequately powered future study.

Dietary adherence was assessed through regular follow-up consultations and participant self-reports, which should be considered when interpreting the findings. Future large-scale multicenter studies are warranted to confirm the observed associations between dietary intervention, serum carotenoid concentrations, and retinal outcomes in patients with AMD.

Lifestyle factors known to influence antioxidant status and AMD risk—including smoking, alcohol consumption, and outdoor/light exposure—were not systematically recorded and therefore could not be accounted for; residual confounding cannot be excluded, and these variables should be prospectively collected in future studies.

Because the intervention was food-based, nutrient delivery depended in part on the seasonal availability of fruits and vegetables; sampling spanned different seasons and was not season-matched, which may have added variability to circulating carotenoid and vitamin concentrations. Future studies should record or standardize the season of sampling.

Because lutein and zeaxanthin co-eluted under the chromatographic conditions applied and were quantified as their sum, the individual contributions and potentially differential responses of each xanthophyll to the intervention could not be resolved; future studies employing chromatographic methods capable of baseline separation of these isomers are warranted.

A further limitation is that macular pigment optical density was not measured; serum concentrations reflect systemic carotenoid status rather than local retinal deposition, and the two are not necessarily concordant. Future studies should combine serum measurements with in vivo assessment of macular pigment (e.g., heterochromatic flicker photometry or dual-wavelength autofluorescence) to directly relate circulating and macular carotenoid status.

## 5. Conclusions

The present study suggests that the intervention was associated with improved serum carotenoid and antioxidant vitamin status in patients with neovascular AMD receiving anti-VEGF therapy. The intervention was associated with significant within-group increases in serum lutein + zeaxanthin, lycopene, and vitamin E concentrations. After adjustment for baseline differences, between-group comparisons were not statistically significant; the numerically larger increases in the intervention group should therefore be regarded as preliminary and hypothesis-generating. Food-based dietary strategies may help modify nutritional biomarkers relevant to retinal health, but confirmation in an adequately powered study is required.

The most pronounced increase in lutein + zeaxanthin concentrations was observed among participants who demonstrated improvement in retinal status. However, retinal outcomes did not differ significantly between groups (Fisher’s exact test, *p* = 0.386 [29]), and some outcome subgroups comprised very few patients (including a single intervention-group patient with retinal deterioration). Any relationship between carotenoid status and retinal outcome is therefore an exploratory, hypothesis-generating observation and does not establish a causal effect. In addition, the observed associations between lutein + zeaxanthin, lycopene, and vitamin A concentrations support the concept that dietary patterns rich in naturally occurring antioxidant compounds may exert complementary and potentially synergistic effects.

These findings support the potential role of dietary management as a valuable adjunct to anti-VEGF therapy and highlight the importance of incorporating nutritional strategies into the comprehensive care of patients with neovascular AMD. Given the exploratory nature of this study, the small sample size, and the single-center design, larger randomized multicenter studies are required to determine whether improvements in nutritional status translate into long-term clinical benefits and retinal outcomes.

Because retinal status was assessed morphologically by OCT/OCTA but visual function (e.g., best-corrected visual acuity) was not analyzed here, and because of the exploratory design, the observed relationship between improved serum antioxidant status and retinal outcome should be regarded as a correlation requiring confirmation in adequately powered, prospective studies incorporating functional and structural clinical endpoints.

## Figures and Tables

**Table 1:** Baseline demographic characteristics and changes in serum concentrations of lutein + zeaxanthin, lycopene, vitamin A, and vitamin E in the control and intervention groups during the study period.

	No Diet (*n* = 21) Baseline ($\bar{x}$ ± SD)	No Diet (*n* = 21) 6 Months ($\bar{x}$ ± SD)	Diet (*n* = 22) Baseline ($\bar{x}$ ± SD)	Diet (*n* = 22) 6 Months ($\bar{x}$ ± SD)
Age (years)	74.5 ± 7.4	–	73.5 ± 8.3	–
Sex, F/M (*n*)	19/2	–	16/6	–
Lutein + Zeaxanthin (mg/L)	0.31 ± 0.61 ^A,B^	0.49 ± 0.69 ^A^	0.51 ± 0.54 ^a,B^	0.87 ± 0.89 ^a^
Lycopene (μmol/L)	0.19 ± 0.15	0.32 ± 0.27	0.20 ± 0.15 ^a^	0.43 ± 0.30 ^a^
Vitamin A [mg/L]	0.29 ± 0.09 ^A^	0.37 ± 0.12 ^A^	0.33 ± 0.13 ^a^	0.40 ± 0.11 ^a^
Vitamin E [mg/L]	12.88 ± 4.47 ^B^	14.87 ± 6.55	10.43 ± 3.85 ^a,B^	12.33 ± 4.59 ^a^

Legend: A—statistically significant differences (*p* < 0.05) in the control group before (day 0) and after 6 months of observation; a—statistically significant differences (*p* < 0.05) in the study group before (day 0) and after 6 months of dietary intervention; B—statistically significant differences (*p* < 0.05) between the study and control groups at day 0.

## Data Availability

The original contributions presented in this study are included in the article. Further inquiries can be directed to the corresponding author.

## Abbreviations

The following abbreviations are used in this manuscript:

AMD	Age-related macular degeneration
VEGF	Vascular endothelial growth factor
AREDS	Age-Related Eye Disease Study
OCT	Optical coherence tomography
OCTA	Optical coherence tomography angiography
LC-MS/MS	Tandem mass spectrometry
TNF-α	Tumor necrosis factor-α
IL-6	Interleukin-6
DNA	Deoxyribonucleic Acid
RPE	Retinal pigment epithelium cells
BHT	Butylated Hydroxytoluene
BMI	Body mass index
WHR	Waist-to-hip ratio
